# Value-impregnated factual claims may undermine medical decision-making

**DOI:** 10.1177/1477750918765283

**Published:** 2018-03-27

**Authors:** Niels Lynøe, Gert Helgesson, Niklas Juth

**Affiliations:** Centre for healthcare ethics, Karolinska Institutet, Tomtebodavägen 18A, 171 77 Stockholm, Sweden

**Keywords:** Autonomy, ethics, personal values, value-impregnated factual claims

## Abstract

Clinical decisions are expected to be based on factual evidence and official values derived from healthcare law and soft laws such as regulations and guidelines. But sometimes personal values instead influence clinical decisions. One way in which personal values may influence medical decision-making is by their affecting factual claims or assumptions made by healthcare providers. Such influence, which we call ‘value-impregnation,’ may be concealed to all concerned stakeholders. We suggest as a hypothesis that healthcare providers’ decision making is sometimes affected by value-impregnated factual claims or assumptions. If such claims influence e.g. doctor–patient encounters, this will likely have a negative impact on the provision of correct information to patients and on patients’ influence on decision making regarding their own care. In this paper, we explore the idea that value-impregnated factual claims influence healthcare decisions through a series of medical examples. We suggest that more research is needed to further examine whether healthcare staff’s personal values influence clinical decision-making.

## Introduction

In modern healthcare, decision-competent patients are expected, and entitled, to get correct and relevant information and be given the opportunity to participate in decision making that concerns their own clinical examinations, treatment, and care. Sometimes such patients also have personal preferences regarding available treatment options.^1^ For instance, some patients may prefer a treatment that provides a better quality of life at the expense of a somewhat shorter life expectancy, compared to another treatment option.^1,2^ Patients’ preferences might, however, differ from those of the healthcare providers, who sometimes think that they know what a certain patient would (or, rather, should) prefer in certain situations. Benevolent healthcare providers might consider what they imagine to be in the patient’s best interest, which might be at odds with what the patient thinks.^1,3^

Providing patients with correct information in a way that does not lead to misunderstandings is especially complicated in cases where the information concerns risk, e.g. risk associated with surgery for aorta aneurism.^4^ Risk information can be framed in ways that influence decision making, for instance by stressing the survival rate of 92% instead of the mortality rate of 8%. Furthermore, using scaring metaphors such as ‘ticking bomb’ or reassuring ones such as ‘routine operation’ might also frame the communication of risks.^4^

The use of such metaphors and descriptions by healthcare personnel may depend on official values as well as on local norms and routines at a specific clinic, or on the healthcare providers’ personal values. Norms and values are more or less intricately integrated with presentations of facts when physicians provide information to patients.^4^ This has been stressed by Molewijk et al.,^4^ who conclude that there is no value-neutral information.

We suggest, however, that it may still be fruitful to separate facts and values by openly discussing framing effects, use of metaphors, and personal values that might distort the decision-making process. In the present study, we will focus on personal values and how they might influence factual judgments. We hypothesize that there are situations where healthcare providers, intentionally or not, *implement their personal values by having them impregnate their factual judgments*. That is to say, situations where their values influence factual judgments so strongly that they in fact determine them (alone or in combination with other factors). By ‘personal values’ we mean values that are contrary to the official ones laid down in healthcare legislation, soft laws, or widely accepted ethical principles, in this case held by the individual healthcare provider or a group of providers. Value impregnation may happen consciously but, we suggest, more often and more likely without the clear awareness of those who let their value judgement be affected by their factual beliefs.

An example of a personal value that runs contrary to official ones is medical paternalism in situations where patients are competent adults.^5^ In a Swedish context, paternalistic actions towards competent patients are unacceptable from the perspective of healthcare law and official regulation. If healthcare providers unconsciously embrace paternalistic attitudes, their values might influence estimations of risk, need, or the patient’s competency or trustworthiness. For instance, if such healthcare providers think that a patient is making an unwise decision, then they might question the patient’s competency and thereby right to have a say regarding his/her own treatment.^6^ The problem of disguised paternalism has been described as ‘smuggling in’ evaluative judgments and ‘masking’ what is clearly *hard paternalism* (deciding without considering the competent patient’s wishes) as a sort of *soft paternalism* (deciding on behalf of the incompetent patient).^7^ What is referred to as ‘masking’ might be related to value-impregnation of factual claims.

From a historical perspective, official values have not always been the ‘right’ ones. Until the law was changed in 1975, sterilisation for family-planning reasons was prohibited in Sweden, as in many other countries.^8^ Sterilisation requests on such grounds were therefore to be turned down. But some physicians were prepared to help women with this kind of sterilisation requests and did so by replacing the indication ‘family-planning’ with ‘medical’ or ‘eugenic’ indication, which were acceptable grounds for sterilisation according to the law.^8^ This kind of proficiency creativity also illustrates how physicians can deliberately mask their personal values by presenting them as factual aspects.

As exemplified above, the official values are sometimes impeding patients from getting what they need. However, sometimes it is the healthcare providers’ personal values that hinder patients from being appropriately met, informed, and treated. In the following we will provide examples of both procedures and show that it is possible to identify cases of value-impregnation of factual judgments. The approach is simple: in cases where the decision pattern of healthcare providers upon scrutiny does not seem explainable from the relevant empirical facts of the case, it is worth considering whether their personal values might explain why decisions were made the way they were. If such values are identified, we suggest that they may be part of the best explanation why healthcare staff made the decisions they did. Finally, we will discuss the reasonableness of our suggestion and other possible explanations.

## The abortion case and judgments of trustworthiness

**Figure 1.**
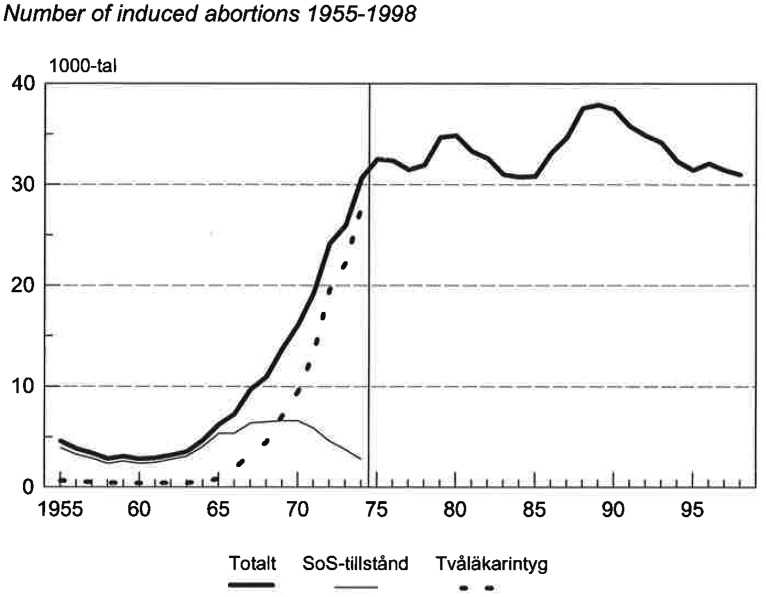
Number of induced abortions in Sweden 1955–2002. The dotted line represents the number of decisions made by the two physicians evaluating the trustworthiness of the abortion-seeking women regarding their descriptions of the social consequences of having a baby. The new liberal Abortion Act was passed in 1974 and came into force 1 January 1975. The figure is from the statistic department of the National Board of Health and Welfare.^10^ ‘Approved by the National Board’ means approved by the National Board of Health and Welfare, and the ‘Two physicians-certificate’ refers to the decision procedure at the time: a gynaecologist and a psychiatrist made the decision.

A study from Sweden describes how physicians denied pregnant women to get an abortion during a period (1946–1964) when it was a legal option to allow abortion on social indication.^9^ During that period, an abortion-seeking woman should be offered an abortion if she made trustworthy claims regarding detrimental social consequences for herself of having the baby. Two physicians, commonly a gynaecologist and a psychiatrist, were to evaluate whether or not the abortion-seeking woman was trustworthy – referred to as the ‘two-physician certificate’ – see Figure 1.^10^ During the time-period 1946–1964, a large majority of all women who sought abortion based on social indication had their request rejected.^9^ Comparing the decisions during 1955–1964 with the radically different decision pattern during the subsequent 10 years (1965–1974), we might ponder whether pregnant women requesting abortion on social indication suddenly became more trustworthy or if something else happened. Estimating trustworthiness is an empirical or factual matter. We suggest that the best explanation for the change in decision patterns is that the evaluating physicians’ personal values influenced their estimations of the women’s trustworthiness, and that either individual physicians changed their values over time or physicians with the old values were replaced by physicians with the new values.

To corroborate our assumption, let us consider Figure 1 and focus on the latter period, 1965–1974.^10^ In 1975, a new liberal Abortion Act was introduced, allowing women to have abortion without being questioned (within certain time-limits). As can be seen from Figure 1, the number of abortions increased considerably during the 10 years before the new liberal abortion act was adopted. The official values laid down in the old Abortion Act remained unchanged during this time-period. Hence, changes in legislation cannot account for the increased number of abortions. Instead, we suggest, *the shift illustrates that a change in physicians’ personal values took place*. Although it cannot be ruled out that women seeking abortion became more trustworthy during 1965–1974, for instance, because they presented more coherent descriptions of their social circumstances, a less farfetched hypothesis is that towards the end of the 1960s, values in society, and also among physicians, changed. The change in values included considerations of women’s right to make their own decisions, a more liberal attitude towards abortion in general, and the aim of promoting women’s reproductive health by avoiding illegal and potentially harmful abortions.

We suggest that this change in values also influenced how physicians estimated the abortion-seeking women’s trustworthiness when describing the social consequences of having a baby and, accordingly, whether or not the social indication was fulfilled.

## Hymen restoration

Another area related to women’s trustworthiness concerns requests of hymen restoration, in situations where the woman fears for her safety due to honour-related threats.^11^ Of course, there is no medical indication for hymen restoration whatsoever, but there may be a social or humanitarian indication for such surgery. There are three factors involved giving rise to such requests. Firstly, in some cultures young women are expected to be virgins when they get married.^11–14^ Secondly, although it is known that approximately 50% of all women do not bleed when they have their first intercourse,^11^ it has been maintained in some cultures that their women have a hymen anatomy that always results in bleeding during their first intercourse.^11,14^ Hence, women from such cultures are expected to produce a red spot on the sheets after the wedding night. Thirdly, some of these women who currently live in Sweden might have had premarital intercourse. When parents arrange a marriage between one of these women and a man from their original culture, the woman may be distressed because she will not produce the expected red spot on the sheets. If she does not, and this is actually controlled, she will be considered a non-virgin and the marriage may be declared illegitimate, resulting in disgrace of the woman’s family. In these circumstances, the only way for the woman’s family to preserve their honour in such a situation may be to expel or kill the woman.^14^ In order to help the woman to produce a red spot on the sheet, healthcare staff could assist in surgically ‘restoring’ the hymen or at least imitate a hymen. This is usually performed by means of a superficial operation, placing a couple of threads which might cause a bleeding at next intercourse. Such a procedure is not illegal but has been strongly criticized.^14^

In a study about GPs’ and gynaecologists’ reasoning about whether or not to perform a hymen restoration for a young woman who desperately requests it, it was found that those who would under no circumstances perform such surgery stated that it did not work and, moreover, that it was unsafe and risky or dangerous.^12^ The same group of physicians also stated that they disbelieved that the young woman’s life was truly threatened. In contrast, respondents who were under certain circumstances prepared to help the woman stated that surgery was an effective, simple, and safe (low risk) way of helping the patient. Furthermore, they stated that it is difficult to question the trustworthiness of the woman regarding the threats.^14,15^ Those who under no circumstances would perform such surgery stated that it would support patriarchal norms, whereas some of those who under certain circumstances would be willing to do it stated that surgery would help undermine patriarchal norms.^12,14^ We think that value-impregnated factual assumptions might be a plausible explanation why some of the physicians responded as they did. Of course, it is possible that the factual beliefs to some extent steer the practical conclusion about hymen surgery to some extent, it seems farfetched to think that trained physicians would have so different beliefs about, for instance, the actual risks of complications regarding such a superficial intervention. A more plausible explanation seems to be that the normative view regarding the intervention steers the factual estimations, i.e. some degree of value-impregnated factual assumptions is taking place.

## Palliative sedation

Swedish palliative care physicians, who rather frequently express ideals regarding dying that oppose ‘an easy way out’, use different practices allowing them to be restrictive regarding deep and continuous palliative sedation, i.e. when the patient is sedated until death.^9^ The restrictive norm on offering sedation becomes manifest in more or less arbitrary rules of thumb, like the one suggesting that deep sedation should not be offered until the last 48 hours before expected death.^9,15–17^ It has also been shown that physicians in general are significantly more liberal regarding their inclination to offer continuous deep sedation compared to the guidelines of palliative care physicians.^18^

Usually patient experiences of unbearable suffering are not questioned, but determining the refractoriness of the symptoms (i.e. whether or not the symptoms are treatable) is the task of the palliative physician.^16,17^ Refractoriness of symptom treatment (i.e. that symptoms are not treatable) is considered an indication for sedation therapy.^16^ If physicians question the refractoriness of a palliative patient’s symptoms, they thereby implicitly question the patient’s trustworthiness when he or she maintains that the symptoms are not treated sufficiently.

In order to illustrate the reasoning, let us take a closer look at two cases told by a palliative care physician (personal communication). The first case concerned a 69-year-old woman suffering from lung cancer that was no longer treatable. She was transferred to a palliative care unit. The patient’s worst symptom was a severe nausea. As symptom treatment she was offered high doses of cortisone, which she initially accepted, and the symptom became tolerable. However, during the treatment she discovered side-effects on her body, such as changes in the shape of her face (moon-face) and other bodily changes. She told the physician that she had always cared about her looks and that it was important to her that her family and friends would not remember her ‘as a monster.’ Therefore she preferred to be continuously deep-sedated until death, without parenteral fluid or nutrition. The patient’s request was rejected for the following reasons: (a) vanity is no reason for sedation therapy and (b) since the symptom (nausea) was not refractory, there was no indication for sedation therapy.

This case can be compared to another case regarding a similarly aged woman suffering from incurable lung cancer. This patient’s dominant symptom was pain, and she was treated with morphine. However, the patient was very interested in literature, and the morphine made her dizzy and unable to concentrate on her reading, so she abstained from morphine during daytime. Although the patient was treated with morphine at night, the pain became more and more intolerable during daytime. This patient too eventually requested continuous deep-sedation therapy until death, without parenteral fluid or nutrition. In this case the physician found that there was an indication for sedation therapy.

The two presented cases are similar regarding the question of refractoriness. Both in the cortisone case and in the morphine case, the symptoms were treatable. In both cases the patients did not accept the side-effects. The only salient difference between the two cases is the patients’ reasons for requesting sedation therapy: preserving looks or preserving intellectual capacity. As far as we can see, the decisions to offer the morphine patient but not the cortisone patient sedation therapy are best explained by reference to the palliative-care physician’s personal values about the importance of appearance and intellectual activity; in the two cases, this influenced the estimation of refractoriness and, accordingly, the indication for sedation therapy. The underlying reasons for the decisions in these cases need not be conscious and intentional. They may be an instance of value-impregnated factual assumptions (Table 1).

**Table 1.** Healthcare providers’ estimations of factual aspects such as trustworthiness, medical risks, medical indication, decision-competency, and classification of medical conditions. ‘Yes’ means that the healthcare providers’ personal values probably influenced their estimations of the factual aspects of the present intervention or clinical examination regarding e.g. patients’ and relatives’ trustworthiness as well as the patients’ decision competency.

	Trust-	Medical	Medical	Decision-	Classification
	worthy	Risks	indication	competency	of conditions
Hymen restoration	Yes	Yes	Yes		Yes
Abortion (1946–1974)	Yes		Yes		Yes
Sedation on request	Yes		Yes	Yes	Yes
Assisted suicide	Yes			Yes	Yes
Shaken baby syndrome	Yes				Yes

## Decision-making capacity

Patients whose preferences deviate from the treatment and care favoured by the treating physician are sometimes considered incapable of autonomous decision-making.^7^ Hermann and colleagues^6^ found a significant variation in how physicians estimated patients’ decision-making capacity in cases where the patient wanted to abstain from life-saving treatment (chemotherapy) and instead preferred assisted suicide. The authors found that the variation in judgment was due to personal values – which they described as paternalistic judgments unconsciously or tacitly coming in ‘through the backdoor.’ The study also showed that 15.9% (n = 143) of the physicians stated that they wanted to be personally convinced that assisted suicide was the best option and used that as a criterion for deeming the patient competent.^6^ Furthermore, 26.1% (n = 166) stated that their own values influenced their judgment of a patient’s decision-making capacity ‘rather much’ or ‘very much’; 51.5% (n = 328) stated that their own values influenced ‘rather little’ and 22.4% (n = 143) stated that their own values had no effect at all on their estimations of patients’ decision-making capacity. These questions, as well as the answers, were provided openly.

Other studies indicate that if questions are provided more indirectly (not openly), the results might be different depending on the situation and on how controversial the issue is.^19^ In one study, Sjöstrand and colleagues^20^ found that psychiatrists were inclined to estimate patients as incompetent when the patients disagreed with the suggested treatment and competent when they agreed to undergo the suggested treatment. This is also supported by the proposal of a new diagnosis when patients in the end of life request euthanasia or continuously deep sedation; they are according to the proposal suffering from ‘demoralization syndrome.’^21^ According to the diagnostic criteria, the wish to die is a cry for help and accordingly such patients are in need of *dignity treatment* and their request for assisted dying should hence not be granted.^22^

These studies indicate that personal values might influence physicians’ judgments of factual issues, see Table 1.

## Truth-telling and ‘shaken baby syndrome’

Child-protection teams who examine suspected child abuse have developed special criteria, used internationally, for identifying parents or guardians suspected of having violently shaken a baby and thereby brought about certain physical damages: subdural hematoma, encephalopathy, and retinal haemorrhage – referred to as ‘the triad.’^23^ When the triad is present, and there are no other ‘acceptable’ explanations, the parent or guardian is suspected to have violently shaken the infant. Child-protection teams have determined what an ‘acceptable explanation’ is.^23^ In a recently published study it was shown that 88% of American physicians who examine infants with suspected shaken-baby syndrome are convinced that if the ‘triad’ is present, then the infant must have been shaken violently.^24^ Hence, if the parent or guardian is unable to provide an acceptable explanation, they are believed to be dishonest and the infant is assumed to have been violently shaken. The problem with this approach is that sometimes the changes in the brain and retina (the triad) might occur due to other explanations, such as birth trauma^25^ or an enlarged circumference of the infant’s scalp.^23^ These phenomena seem to be associated with an increased risk of spontaneous bleeding in the brain and retina. Such bleedings may also be caused by a minor fall.^23^

Parents may lie in order to protect themselves from prosecution, but they may also tell the truth. When using the criteria of child-protection teams for identification of shaken-baby cases, there is a risk that innocent parents are prosecuted and convicted, in particular since members of child-protection teams are also used as expert witnesses by the police and by prosecutors.^26^ Epidemiologists found that the incidence of homicide among infants sharply increased after 1980 (until 2005) from a stable incidence during the period 1940–1979.^27^ The authors suggested that the classification of homicides and accidental deaths in recent decades has been influenced by ethical considerations relating to the protection of infants from abuse, rather than by scientifically based considerations of what is reasonable proof of abuse.^27^

The child-protection teams, however, maintain that their criteria are scientifically evidence-based.^28,29^ But the studies behind the evidence are not independent of the assumptions made by the child-protection teams; instead, they have been used by researchers when classifying study cases and controls resulting in the disappearance of false-positive cases. This in turn has impact on the calculation of the positive predictive value of the diagnostic accuracy, which becomes 100%.^30^ Hence, the results from most of these studies are based on circular reasoning.^23,30^

We suggest that also the classification of study cases and controls made by child protection teams is the result of value-impregnation of factual aspects. The values impregnate the choice of theory and the derived criteria when classifying study-cases and controls, which in turn influences estimating the trustworthiness of the infants’ parents.

## Discussion

A common denominator to several of the presented cases is that patients request a treatment that is considered inappropriate by healthcare providers. We suggest that in cases where the personal values of the healthcare provider strongly influence the situation, the reaction might be to question the patients’ or relatives’ trustworthiness when describing concerns, symptoms, and sufferings. Even the evaluation of ‘medical indication’ sometimes seems to be based on physicians’ personal values in relation to patients’ requests.^31^ There are some indications that the more controversial an issue is, the stronger the influence of the physicians’ own values on their decisions.^19^

### Why do healthcare providers mask personal values?

Why are healthcare providers not explicit about their personal values and rather have them influence the decision-making process ‘through the backdoor’? Why do they not just present their personal values openly? One answer might be that they are not always aware that they embrace personal values that disagree with official values, simply because they are not aware of the values they have. An open discussion about the values of healthcare could perhaps promote such awareness.

Another answer might be that they do not want to be open about their personal values. Healthcare providers in Sweden are well aware that they are expected not to let their personal values influence clinical decision-making – they are expected to act in accordance with healthcare legislation and official guidelines. There is no room for conscientious objections in the Swedish healthcare system. Healthcare providers are furthermore expected to provide correct and relevant information to competent patients and invite patients to make shared decisions in accordance with the principle of patient-centred care. If healthcare providers are aware of their values and want to influence the outcome in a certain direction, then being tacit about them and letting their values influence factual and empirical statements may be the best way to succeed, in particular in relation to patients, where healthcare providers have the information advantage. This will, of course, be a piecemeal approach since it is only directed at influencing individual cases and not the general approach to the issues they feel strongly about.

### Are official values always progressive?

Several of our cases concern the contrast between progressive official values, to a great extent expressed in legal regulations, and stale private values held by healthcare providers. They capture a discrepancy that may be, at least partly, explained by the considerable changes in the healthcare system over a series of decades, where patient autonomy, patient-centredness, and shared decision-making have become increasingly stressed. Perhaps not all healthcare providers have embarked upon the same journey towards increased attention to and participation by the individual patient.

However, our main point is personal values may influence factual judgments, with the potential effect that patients are not properly informed, or get excluded from the decision process altogether, not that deviations from public values always lead to negative consequences for individual patients. There is generally something problematic about deviating from official healthcare values, since a discrepancy between official values and practice is likely to lead to unequal treatment of equal cases. But it cannot be excluded beforehand that official healthcare values do not adequately consider patients’ health-related needs, and that individual healthcare providers disagreeing with these values provide more adequate care.

More research is needed both regarding discrepancies between official healthcare values and values held by healthcare providers and regarding how to reduce the number of events where personal values override the official values of healthcare. Along with this, we would like to see a vivid debate regarding the values that are and should be promoted and protected by healthcare.

## Conclusion

We have analysed how value-impregnation might influence healthcare providers’ judgments of factual aspects, such as patients’ trustworthiness, estimations of risk, presence of medical indication for specific treatments, and patients’ decision-making capacity. If our analysis is reasonable and value-impregnation is a phenomenon that might undermine shared decision making and patient involvement, then the phenomenon deserves to be further studied.
